# Editorial: Revisiting mouse models of traumatic brain injuries: a focus on intracellular mechanisms

**DOI:** 10.3389/fncel.2024.1505348

**Published:** 2024-10-17

**Authors:** Saad Omais, Firas Kobeissy, Kazem Zibara, Abdullah Shaito

**Affiliations:** ^1^Department of Biological Sciences, School of Arts and Sciences, Lebanese American University, Beirut, Lebanon; ^2^Department of Neurobiology, Center for Neurotrauma, Multiomics and Biomarkers, Morehouse School of Medicine, Atlanta, GA, United States; ^3^PRASE and Biology Department, Faculty of Sciences-I, Lebanese University, Beirut, Lebanon; ^4^Biomedical Research Center, Department of Biomedical Sciences at College of Health Sciences, and College of Medicine, Qatar University, Doha, Qatar

**Keywords:** traumatic brain injury, mouse models, intracellular mechanisms, *in vivo*, basic-to-translational research, reproducibility

Traumatic brain injury (TBI) continues to be a critical health condition, especially for the newly born, the elderly, and victims of motor vehicle accidents, sports concussions, war blasts, etc. Due to the extremely complicated nature of primary and secondary sequelae following injury and the complexity of research on the human brain, little can be inferred from human clinical studies concerning TBI pathogenesis and progression (Maas et al., 2017). Instead, mouse models have overwhelmed the *in vivo* research on TBI. Drivers for the dominance of TBI studies in mice range from minimally serious ethical concerns of experimenting with the mouse brain, to the homogeneous genetic background of mouse strains, to the advantages of employing a larger statistically relevant sample size. Studies on rodents are nonetheless burdened with several limitations, such as non-standardized categorization of severity, use of neuroprotective anesthetics, and a different time-course of progression post-TBI damage compared to humans (Siebold et al., 2018; Radabaugh et al., 2023). More importantly, short- and long-term dysregulation of neuronal and glial cells at the perilesional region are often neglected at the expense of studying biomarkers that are more readily detected in the bloodstream (Sabirov et al., 2022).

This Research Topic aimed to highlight the relevance and shortcomings of existing TBI mouse models (CCI, FPI, blast TBI, CHIMERA, etc.) and to investigate the intracellular mechanisms dysregulated in the injured brain (Petersen et al., 2021; Xiong et al., 2013). The dysregulated processes could include rapid short-term changes of molecular signatures or persistent long-term sequelae such as those linked to neurodegeneration. Moreover, several studies in mice have attempted pharmacological and neurotherapeutic interventions that can ameliorate TBI defects, but these await the proper assessment of their potential translation into human studies (Kabadi and Faden, 2014). There are also recent methodological interventions that can expand our knowledge of how distinct cell populations are involved in TBI etiology, progression, and treatment. As such, a reference addressing these issues would help resolve much of the concerns on reproducibility and translation of basic molecular phenomena into the clinical manifestation of TBI.

In a comprehensive review, Fesharaki-Zadeh and Datta highlighted the various TBI animal models, including drosophila, mice, and larger animals (e.g., sheep and primates). They describe the extent to which these models mimic TBI pathology, including cortical injury, compromise of the blood-brain barrier, hemorrhage, and various motor and cognitive behavioral deficits. More importantly, the authors discussed the advantages and limitations of each model in light of recent studies. They have also alluded to the experimental models studying the combination of stress and TBI. Their review additionally focuses on signatures of long-term neuronal dysregulation post-TBI, such as Tau hyperphosphorylation, calcium dysregulation, and mitochondrial dysfunction. Overall, this review provided an up-to-date overview of existing TBI models, setting the stage for testing much-needed new TBI therapies.

Chronic sequelae to TBI were the subject of the study by Zuckerman et al.. They subjected rTg450 HEMI mice (known to overexpress human tau and mimic symptoms of frontotemporal dementia) to an open-field low-intensity blast (LIB), mimicking the persistent brain damage following mild TBI. The group employed the CognitionWall test system to assess cognitive deficits pertaining to learning tasks. Their results reveal high levels of complexity and heterogeneity in learning flexibility post-injury. They also established a novel individualized method to evaluate the capacity of the individual mice for reversal learning. They established that mice exhibiting a “learning deficit” or a “mild learning deficit” could still manifest different learning patterns within the same group. This kind of thorough and personalized assessment could help resolve the sources of heterogeneity in human TBI patients.

The neuroinflammatory cells are crucial contributors to secondary responses following neurotrauma. Abou-El-Hassan et al. offer an update of the recent methodological approaches to study the role of neuroinflammatory cells in TBI. They discuss the techniques used to address TBI neuroimmunology, including adoptive cell transfer (ACT), direct intra-CNS injections, cell depletion therapy, genetic engineering, molecular imaging, and *in vitro* co-culture systems. For instance, the use of ACT revealed that T-cells play dual roles, dependent on their subtypes, in exacerbating or ameliorating inflammation post-TBI. This manuscript highlights the complexity of studying the cellular correlates of TBI which leads to heterogeneous effects that need to be taken into account when modeling TBI in rodents.

In a new study, Pischiutta et al. introduced a 3D *in vitro* model of brain contusion consisting of an organotypic cortical brain slice in mice. One week after the slices were subjected to a focal CCI impact through an electromagnetic impactor, the culture model manifested a TBI-like phenotype, including a temporal and spatial increase in cell death, a decline in neuronal arborization, marked astrocytic damage, and microglial activation. Through systems biology analysis, altered proteins in the cell culture model matched known alterations of TBI biomarkers. Finally, delivery of mesenchymal stem cells (MSC)-secretome 1 h after injury to the cultured slices protected from neuronal loss following injury. This study reveals the success of inducing TBI *in vitro*, recapitulating the hallmark signatures of TBI in mice, and suggests a therapeutic potential of MSCs secretome in TBI.

By looking at cortical mouse neurons subjected to mechanical injury *in vitro*, Li et al. tested the effect of Transforming Growth Factor β1 (TGF-β1), known to exhibit neuroprotective roles in TBI in addition to its known role in reducing inflammation and promoting synaptogenesis. Cultured neurons subject to trauma showed increased levels of cell death (marked by LDH release and TUNEL staining) and elevated levels of autophagy-related genes (confirmed by RNA-seq, Western Blot, and immunohistochemistry). Both phenotypes were rescued, partially or entirely, following TGF-β1 treatment, implicating TGF-β1 in apoptotic and autophagic cell death pathways. These data support the use of TGF- β1 in treating TBI pathology, pending clinical trials.

The Research Topic also includes a review of the non-pharmacological approaches to manage TBI. Wu et al. comprehensively reviewed the evidence for the role of acupuncture in animal studies of TBI. They included 14 original studies assessing manual acupuncture (nine studies), electroacupuncture (four studies), or bloodletting puncture (one study). The review detailed how acupuncture targets different cellular populations (i.e., microglia and astrocytes) and rescues defects in molecular pathways (autophagy and apoptosis) disrupted by neurotrauma. The review pinpoints several limitations, such as the scarcity of clinical trials on the utility of acupuncture in TBI. Altogether, given the lack of approved treatments for TBI, this review makes the case for broadening the scope of rehabilitative approaches toward alternative modalities such as acupuncture to improve functional recovery after injury.

In conclusion, research into TBI mouse models is accelerating on all levels, whether by looking for potential diagnostic biomarkers, investigating short- and long-term damage post injury or testing therapeutics to enhance recovery. The special topic at hand discussed these issues, with an emphasis on underlying intracellular mechanisms, in hopes of moving closer toward meaningful and relevant translational outcomes.
